# ENGINEERED NANOBODIES WITH PROGRAMMABLE TARGET ANTIGEN PROTEOLYSIS (PTAP) FUSIONS REGULATE INTRACELLULAR ALPHA-SYNUCLEIN IN VITRO AND IN VIVO

**DOI:** 10.21203/rs.3.rs-4088206/v1

**Published:** 2024-03-28

**Authors:** Diptaman Chatterjee, Lianna Y. D’Brant, Benjamin M. Hiller, David J. Marmion, Ivette M. Sandoval, Kelvin C. Luk, Fredric P. Manfredsson, Anne Messer, Jeffrey H. Kordower, David C. Butler

**Affiliations:** 1 Department of Neurological Sciences, Rush University Medical Center, Chicago, IL 60612; 2 Department of Neurology, Feinberg School of Medicine, Northwestern University, Chicago, IL 60611; 3 Regenerative Research Foundation, Neural Stem Cell Institute, Rensselaer, NY 12144; 4 Department of Translational Neuroscience, Barrow Neurological Institute, Phoenix, AZ 85013; 5 Department of Pathology and Laboratory Medicine, University of Pennsylvania, Philadelphia, PA 19147; 6 Department of Biomedical Sciences, University at Albany, Albany, NY 12208; 7 ASU-Banner Neurodegenerative Disease Research Center and School of Life Sciences, Arizona State University, Tempe, AZ 85281

## Abstract

Alpha-synuclein (αSyn) aggregation and the formation of Lewy pathology (LP) is a foundational pathophysiological phenomenon in synucleinopathies. Delivering therapeutic single-chain and single-domain antibodies that bind pathogenic targets can disrupt intracellular aggregation. The fusion of antibody fragments to a negatively-charged proteasomal targeting motif (PEST) creates bifunctional constructs that enhance both solubility and turnover. With sequence-specific point mutations of PEST sequences that modulate proteasomal degradation efficiency, we report the creation of Programmable Target Antigen Proteolysis (PTAP) technology that can provide graded control over the levels of target antigens. We have previously demonstrated our lead anti-αSyn intrabody, VH14-PEST, is capable of reducing the pathological burden of synucleinopathy *in vitro* and *in vivo*. Here, we report a family of fully humanized VH14-PTAP constructs for controllable, therapeutic targeting of intracellular α-Syn. In cells, we demonstrate successful target engagement and efficacy of VH14-hPEST intrabodies, and validate proof-of-principle in human cells using 3D human organoids derived from PD-patient induced pluripotent stem cells (iPSC). In two synuclein-based rat models, PTAP intrabodies attenuated nigral αSyn pathology, preserved nigrostriatal dopaminergic tone, and slowed the propagation of αSyn pathology. These data demonstrate the potency of intracellular αSyn targeting as a method to alleviate pathology and highlight the potential clinical utility of PTAP intrabodies.

## Introduction

Intracellular aggregation of the intrinsically disordered protein, alpha-synuclein (αSyn), is the pathological foundation for synucleinopathy-based neurodegenerative diseases, such as Parkinson’s disease (PD), Multiple systems atrophy (MSA) and Lewy Body dementia (LBD)^1^. Genetic evidence from familial PD studies link rare duplications^2,3^ and triplications^4^ of the *SNCA* gene encoding αSyn as causative factors, indicating that when αSyn is highly elevated it is pathogenic. Other mutations in the *SNCA* locus have also been identified in inherited PD^5–11^, and linkage to SNCA has been shown for LBD^12^, augmenting the protein’s role in pathophysiology. Additionally, perturbed αSyn protein regulation, usually as a result of encumbered or deficient proteostatic mechanisms, can lead to αSyn accumulation and toxicity^13–15^, highlighting a threshold between monomeric αSyn and proteopathic aggregates. Since its discovery as the primary proteinaceous constituent in Lewy pathology^16^ and Papp-Lantos bodies^17^, a growing body of evidence has demonstrated numerous cellular dysfunctions and toxicities associated with αSyn aggregation^18^.

Several pre-clinical efforts have focused on therapeutics to abrogate αSyn levels or reduce αSyn aggregation, using a range of nucleic acid and protein approaches^19,20^. Intracellular antibody fragments, either single-domain nanobodies or single-chain variable fragment (scFv) intrabodies, offer specificity of antibody binding with molecular engineering options to redirect and program the optimal αSyn levels within the cell^21^. An intrinsically soluble scFv was selected from a screen of binders to the NAC domain of αSyn^22^; this construct can down-regulate αSyn protein^23^ and improve locomoter behavior when delivered using AAV in a rodent synucleinopathy model^24^.

Single-domain nanobodies offer additional advantages for engineering and delivering intrabodies, due to their smaller size and options for multifunctional fusions^25^. We have previously shown that the bifunctional human nanobody, VH14, fused to a proteasomal targeting peptide (PEST) is capable of alleviating pathology and providing functional and structural rescue of nigrostriatal dopamine in an intranigral αSyn overexpression model of PD^26^. This approach offers combinatorial mechanisms of action by both intrabody interference with the aggregation-contingent NAC domain and promotion of αSyn turnover via proteasomal clearance. In our previous report, VH14-PEST was capable of significantly reducing nigral phospho-serine 129 (pS129) αSyn while not significantly ablating total αSyn signal^26^.

Fusions of the C-terminal PEST degron derived from murine ornithine decarboxylase (mODC) enhance intrabody solubility while facilitating intrabody-antigen turnover^27,28^. In the present study, we further develop and engineer VH14 for clinical use and generate a series of constructs for the targeted regulation of intracellular αSyn levels. We first validate that intrabody fusions to human ODC PEST (hPEST) degron provide equivalent levels of αSyn turnover compared to the initially characterized murine PEST (mPEST). Further mutagenesis of the hPEST sequence was used to create degron variants with differing levels of clearance of the target. Upon fusion to VH14, hPEST degron variants demonstrated graded reductions in intracellular αSyn in the range of 30–60% of control levels in a rat striatal cell line. We refer to this technology as Programmable Target Antigen Proteolysis (PTAP).

Using induced pluripotent stem cell (iPSC)-based 3D organoids derived from a PD patient with a triplication of the *SNCA* locus (3X-*SNCA*), we validated that expression of anti-αSyn PTAP intrabodies in 60-DIV forebrain organoids can effectively engage human αSyn. Finally, we provide proof-of-concept of the efficacy of PTAP intrabodies *in vivo* in two separate model paradigms of synuclein pathology. We show that AAV-delivered PTAP intrabodies can modify αSyn pathology in rat models of PD and demonstrate mitigation of spread and progression of synucleinopathy when administered broadly throughout the CNS. Collectively, these findings describe a novel approach to long-term titration of intracellular αSyn levels and substantiate the use of fully human VH14-hPEST and PTAP degron variants for continued pre-clinical development for the intracellular treatment of synucleinopathies.

## Results

### Human PEST sequences demonstrate equivalent degradation efficiencies as murine PEST

**Figure 1.**
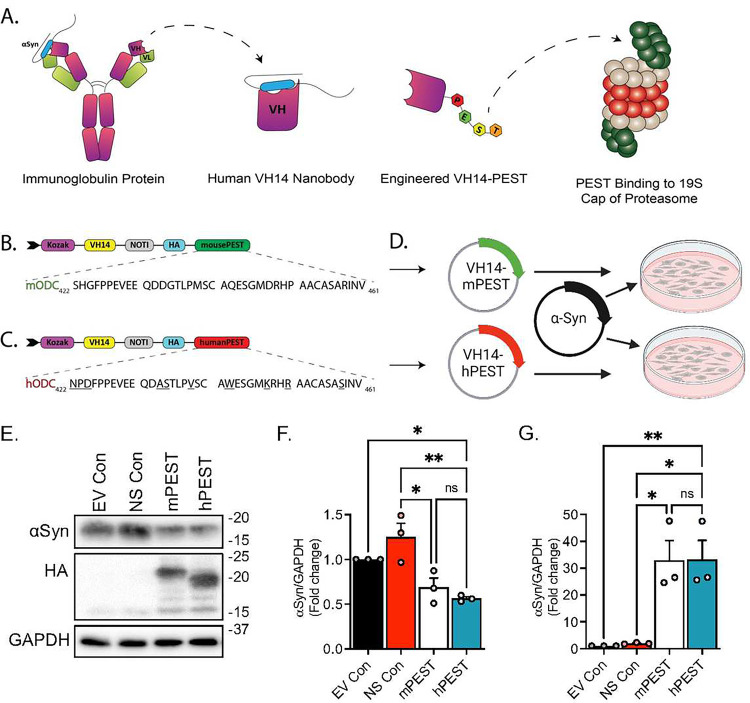
VH14-hPEST-mediated αSyn turnover is equally efficient to that of VH14-mPEST. A) Schematic of nanobody isolation and engineering. VH14 nanobody is single heavy-chain domain from a human repertoire that binds to the NAC domain of αSyn. The fusion of the PEST peptide enhances construct solubility and adds functionality through the shuttling of cargo to the proteasome for degradation. B) Sequence comparison of the C-terminal PEST degron from murine ornithine decarboxylase and C) human ornithine decarboxylase. D) Study design: HEK293 cells were co-transfected with αSyn and either empty vector control (CON), intrabody without start codon (NS), VH14-mPEST, or VH14-hPEST. E) Corresponding immunoblot data demonstrated reductions in αSyn protein expression with both murine and human PEST constructs. F) Densitometric analysis of immunoblot illustrating degree of αSyn signal across mPEST and hPEST treatment groups. G) Densiometric analysis of intrabody (HA) expression. (Statistics: All *p<0.05, **p<0.01).

The initial objective of this study was to generate a fully human construct of the nanobody VH14-PEST for experimentation and the potential clinical development in the targeting of human αSyn. Although VH14 is a single-domain human binder selected from a yeast display library (Fig. 1a)^22^, our initial proof-of-principle characterizations were performed with a murine PEST degron (Fig. 1a)^26,28^, thus warranting testing of PEST sequences derived from the C-terminus of human ODC (hODC). hODC_441–462_ shares ~75% sequence homology with that of its murine counterpart (Fig. 1b,c). We co-transfected HEK293 cells with plasmids expressing human αSyn and either the previously characterized hybrid VH14-mPEST or the fully humanized VH14-hPEST (Fig. 1d). Immunoblot analysis revealed a significant reduction in human αSyn levels following VH14-mPEST and VH14-hPEST treatments for 72 hours (p<0.05, p<0.01, respectively), compared to the control vector intrabody without a start codon control (NS) (Fig. 1e,f). Intrabody protein expression (HA) was not observed in either the empty vector control or NS control treated cells (Fig. 1e). As anticipated, VH14-hPEST and VH14-mPEST did not show a significant difference between each other (Fig. 1g).

A parallel set of experiments was conducted in murine ST14A immortalized cells to confirm a conserved mechanism of action across species with the PEST degron. We co-transfected ST14A cells with plasmids expressing αSyn tethered to GFP with a flexible (Gly_4_Ser)_4_ linker (αSyn~GFP) and either VH14-mPEST or VH14-hPEST (Fig.S1a,b,c). Immunofluorescent images of the cells 48 hours post-transfection show marked decreases in αSyn~GFP signal in both VH14-mPEST and VH14-hPEST-treated cells compared to naïve control cell populations (Fig.S1d). Corresponding immunoblots probed for total αSyn~GFP validate the observed reductions in immunofluorescence; thus, VH14-hPEST demonstrates equivalent αSyn~GFP turnover efficiency as compared to its hybrid counterpart, compared to control expression levels (Fig.S1e).

To confirm that αSyn lowering effects of the VH14-hPEST are mediated through the proteasome, we co-transfected HEK293 cells with αSyn~GFP and either VH14-hPEST or B8-hPEST, a control intrabody raised against botulinum neurotoxin light chain, an absent antigen within our model system (Fig.S2a,b)^29^. Transfected cells were then treated with Epoxomicin (Epox; 10 μM, 16 h), a selective inhibitor of the 26S proteasome, which was used to examine the extent to which turnover of the VH14-hPEST is proteasome-mediated. In agreement with our previous studies, showing that VH14-mPEST and αSyn~GFP are degraded via the proteasome^27^, VH14-hPEST significantly (p < 0.0001) lowered αSyn~GFP expression compared to control intrabody B8-hPEST (Fig.S2c,d). These results were confirmed by quantitative western blotting (Fig.S2e). Conversely, treatment with Epox, significantly increased αSyn~GFP expression by live cell imaging (p < 0.01) (Fig. S2c,d) and western blot analysis (p < 0.01) (Fig.S2e) compared to VEH (DMSO) control. Intrabody levels, measured by HA, were minimally detected in VEH-treated cells (Fig.S2f); However, following treatment with Epox, VH14-hPEST and B8-hPEST intrabody levels were significantly increased (p< .0001) (Fig. S2f).

### Targeted amino-acid substitutions in the proteasome-association domain of PEST sequence can modulate proteasomal turnover of intracellular αSyn

**Figure 2.**
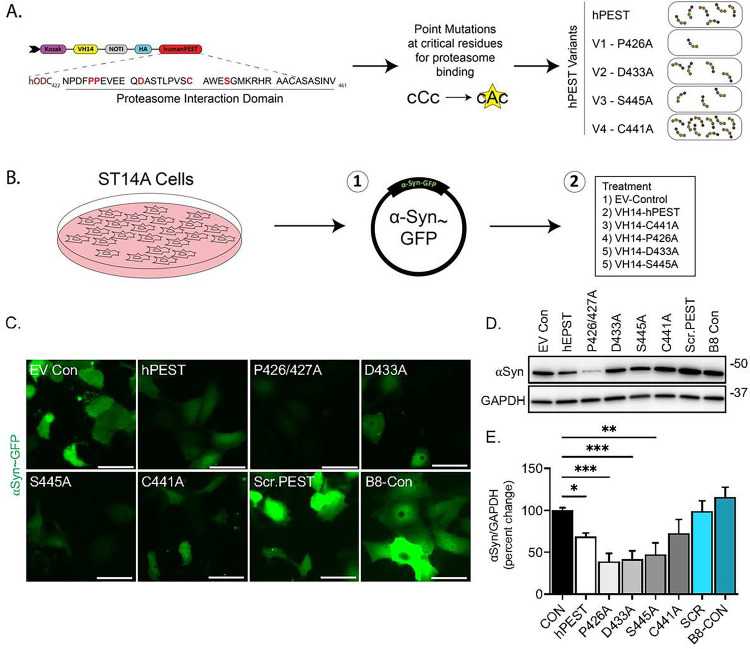
Engineered mutations in hPEST sequence at residues critical for proteasome binding alter degradation efficiency of PEST motifs. A) Human PEST sequence with highlighted amino acids designating conserved residues from murine PEST. Schematic indicates experimental design for alanine-substitutions to generate hPEST variants. B) Study design: ST14A cells were co-transfected with αSyn~GFP and VH14 intrabody plasmids with hPEST variant fusions. C) Fluorescent readouts of native GFP indicating αSyn expression levels, illustrating graded αSyn signal reduction in intrabody treated cells. (Scale bar – 50μm). D) Immunoblot data from ST14A lysates validate reductions in αSyn protein expression with differential capacities for αSyn degradation. E) Densitometric analysis of immunoblot illustrating degree of αSyn signal across hPEST variant treatment groups. (Statistics: All *p<0.05**, p<0.01, ***p<0.001, ****p<0.0001).

Conserved amino acid residues between hPEST and mPEST sequences (Fig. 2a) suggest key proteasomal association domains enhance ubiquitin-independent degradation. PEST-induced protein turnover requires two factors for ubiquitin-independent degradation signaling: 1) a disordered association interface with the 19S cap subunit of the 26S proteasome and 2) sufficient peptide length from the core protein to permit proteasomal entry^30–32^. PEST sequence mutations within the proteasome association domain have been shown to ablate proteasomal localization and extend protein stability and half-life^31^. Using this principle, we engineered targeted point mutations at conserved murine/human PEST residues critical for proteasomal binding to modify efficiency of proteasomal targeting at D433A, C441A, S445A, and P426A/P427A. We generated a series of targeted alanine substitutions at homologous residue binding sites (Fig. 2a), including C441, within the proteasome interacting domain: D433A, C441A, S445A, and a double-mutant at P426A/427A (P426A) (PTAP variants).

To establish the contribution of PEST variants to the proteasomal turnover efficiency of tagged proteins, we utilized an identical screening platform as reported in the validation of hPEST (Fig.S1). ST14A cells were co-transfected with plasmids expressing αSyn~GFP and VH14 tagged with PTAP variants, or the parent VH14-hPEST intrabody (Fig. 2b). Immunofluorescence images of αSyn~GFP at 72-hours post-intrabody transfection depict a gradation of αSyn expression levels across VH14-PTAP variants, with the lowest signal observed in the P426A treated cells compared to control αSyn~GFP expression (Fig. 2c). Corresponding immunoblot expression analysis of total αSyn confirmed a reduction in αSyn protein by PTAP variants compared to control treated cells (Fig. 2d). Quantification of αSyn expression displays statistically significant comparative reductions of ~31% (p<0.05, VH14-hPEST), 58% (p<0.001, D433A), 52% (p<0.01, S445A) and 61% (p<0.001, P426A) compared to control αSyn expression (Fig. 2e). This validates that PTAP fusions to the human VH14 intrabody are able to titrate intracellular αSyn levels in 2D neuron cultures.

### Fully humanized VH14-hPEST and PTAP intrabodies engage human αSyn and attenuate αSyn levels in patient-derived 3X SNCA forebrain organoid cultures

**Figure 3.**
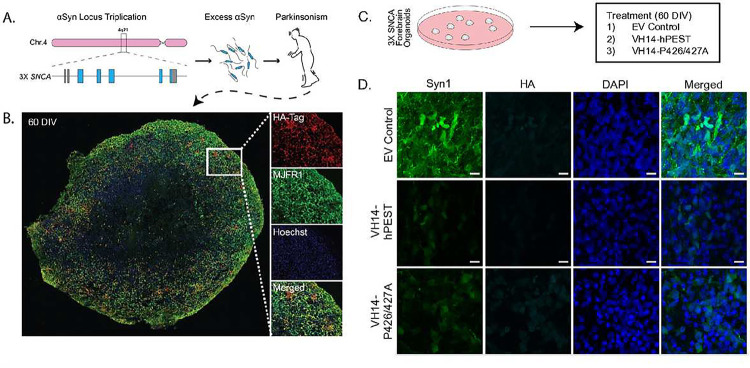
VH14-PTAP intrabodies modify αSyn levels in human iPSC organoids derived from PD patient with *SNCA* triplication. A) Diagram detailing *SNCA* locus from PD patient with triplication of αSyn gene. B) iPSCs derived from patients are grown and patterened into forebrain 3D organoids that can be infected with lentivirus expressing intrabodies long-term. Representative image of a post-LV-VH14-hPEST treated organoid grown for 60 days *in vitro* (HA-tag = intrabody, MJFR1 = human αSyn). C) Experimental design for testing PTAP intrabodies in forebrain organoids. D) Immunofluorescent labeling of hu-α‐syn levels in organoids infected with lentivirus carrying VH14-PTAP intrabodies shows reduction in hu-α‐syn signal compared to EV-treated organoids.

As our ultimate objective is to characterize αSyn-targeting intrabody therapy for clinical use, we next aimed to determine whether VH14-hPEST or PTAP degron variants are effective in human cell-based systems. Additionally, we wanted to generate a screening system with endogenous αSyn expression for testing intrabody efficacy. To that end, we developed 3D organoid cultures derived from 3X-*SNCA* patients with PD (Fig. 3a,b)^33^. Using iPSCs derived from these patients and a control iPSC line (Fig. 3a,c), we generated embryoid bodies and patterned cortical organoids to differentiate into a forebrain lineage, as described in Bowles et al, 2021^34^. At 20 days *in vitro* (DIV), forebrain organoids stain positively for forebrain markers Sox2, Pax6, FoxG1, and show no staining for the negative selection marker Sox10, indicating authentic forebrain lineage and development (data not shown).

3X-*SNCA* organoids were stably transduced at 30 DIV with lentiviral constructs expressing VH14-hPEST, VH14-P426A, or EV-Control to evaluate target engagement and reduction of αSyn protein expression (Fig. 3). At 60 DIV, intrabody treated organoids stained positive for intrabody construct expression when assayed 30 days post-transduction, as indicated by staining of the HA-tag incorporated into intrabody sequences (Fig. 3b,c). Compared to empty control EV-treated organoids, VH14-hPEST and VH14-P426A-treated organoids showed notable decreases in αSyn levels in HA+ neurons, indicating VH14-hPEST-mediated target reduction (Fig. 3d). Although organoids treated with PTAP intrabodies did not show statistically significant reductions in total αSyn protein by western blot analysis (Fig.S3a,b), 3X-SNCA organoids treated with intrabodies showed a marked reduction in TUNEL staining, indicating lower rates of cell death compared to EV-control treated 3X-SNCA organoids (Fig.S1c,d). These data suggest intrabodies can be delivered effectively to human neurons and engage endogenous human αSyn.

### Intranigral AAV-VH14-hPEST reduces phosphorylated αSyn pathology and protects nigrostriatal neurons from αSyn-mediated neurotoxicity *in vivo*

**Figure 4.**
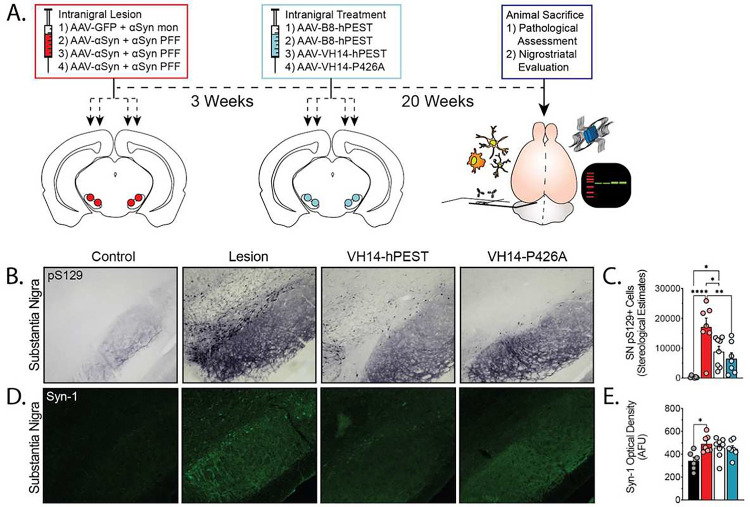
Intranigral treatment with AAV-VH14-PTAP intrabodies attenuates αSyn pathology and mitigates neuroinflammatory phenotypes. A) Study design: Aged rats received bilateral dual intranigral lesions of low-titer AAV-αSyn and αSyn PFFs, or control injections. Three weeks post-lesion, animals received injections of AAV carrying VH14-hPEST, VH14-P426A, or B8-hPEST control. Animals were sacrificed after 20-weeks post-treatment. B) Microphotographs of pS129+ stained αSyn pathology in the SN. C) Corresponding stereological estimations of pS129+ aggregates in the SN. D) Images of immunofluorescently labeled tissue stained for total αSyn and E) densitometric analysis of αSyn signal in the SN. F) Immunoblot analysis illustrating total αSyn expression from midbrain lysate preps. (Statistics: All *p<0.05, **p<0.01, ***p<0.001, ****p<0.0001).

To establish whether αSyn-targeting PTAP intrabodies are effective *in vivo*, we first utilized an experimental paradigm to directly test intrabody therapeutic efficacy at the site of αSyn aggregation. For the primary *in vivo* experiments, we utilized an adapted intranigral, dual-hit rat model of synucleinopathy that induces rapid αSyn pathological aggregation and causes a substantial neuroanatomical deficit in the nigrostriatal system (Fig. 4a)^35,36^. Towards this end, we bilaterally injected a mixed injectant of low-titer adeno-associated virus (AAV)-αSyn plus pre-formed αSyn fibrils (PFFs) into two targets within the SN of adult rats (Fig. 4a). To optimize seeding, we analyzed EM images of PFFs to ensure a majority of fibrils were <50 nm in length prior to surgery (Fig.S4a,b)^37–39^. Control animals received injections with equivalent concentrations of αSyn monomers and AAV-GFP. After 3-weeks post-lesion, we administered treatment vector into the same nigral coordinates (Fig. 4a). Lesioned animals were treated with VH14-hPEST, VH14-P426A, or the B8-hPEST intrabody^29^. Control subjects also received injections of B8-hPEST to account for viral transduction and protein expression. Intrabody treatments resulted in no significant changes in animal body weight (Fig.S4d) and no observable issues in home-cage behavior. Animals were sacrificed 5-months post-treatment for histopathological and molecular assessments (Fig. 4a).

We first conducted pathological assessments of αSyn employing stereological estimates of pS129+ aggregates in the substantia nigra (Fig. 4b). As expected, lesioned animals treated with the B8-hPEST intrabody showed significant pathology throughout the SN and into neighboring nuclei (17124±7907 pS129+ aggregates) compared to AAV-GFP/monomer control subjects that displayed negligible pathology (336±331 pS129+ aggregates, p<0.0001) (Fig. 4c). Both VH14-hPEST and VH14-P426A significantly reduced pS129 immunoreactivity, with VH14-hPEST showing a 48.1% reduction (8883±4865 pS129+ aggregates, p<0.05) and VH14-P426A providing greater reduction at 61.9% (6525±5096 pS129+ aggregates, p<0.01) although the two treatment groups were not statistically different from each other (p>0.05) (Fig. 4c).

To quantify the effect on total αSyn levels, we stained sections with a pan-αSyn marker (Syn-1) and densitometrically evaluated staining intensity (Fig. 4d,e). Lesioned animals all displayed pronounced increases in αSyn immunoreactivity, with densely stained cell bodies most evident along the nigrostriatal tract in B8-hPEST treated animals (Fig. 4d). Signal intensity in B8-hPEST-treated αSyn-lesioned animals showed a statistically significant 43.5% increase in αSyn signal compared to control animals (p<0.05, Fig. 4e). However, we did not observe statistically significant decreases in total αSyn signal as a result of VH14-PTAP treatments compared to B8-hPEST-treated lesioned animals (Fig. 4e). No statistical differences were observed between control animals and either VH14-hPEST or VH14-P426A (Fig. 4e). These data indicate the level of αSyn reduction from VH14-PTAP intrabodies does not significantly ablate signal beyond control levels of expression in this model system. To confirm, we evaluated midbrain αSyn reduction in intrabody-treated cohorts by immunoblot (Fig.S4e). Immonoblots for αSyn show modest reductions in total αSyn signal in VH14-hPEST and VH14-P426A-treated animals compared to control and lesioned animals, although with minimal evidence of differences in αSyn levels between the two intrabody treated cohorts (Fig.S4e).

**Figure 5.**
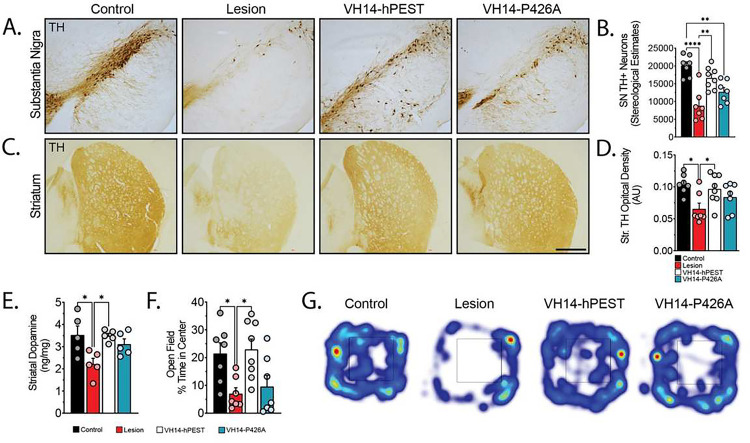
VH14-hPEST provides structural and functional nigrostriatal protection against αSyn insult. A) Representative microphotographs of TH-ir dopaminergic neurons in the SN and B) corresponding stereological estimates of SN TH+ neurons. C) Representative images of striatal terminals labeled with TH. D) Densitometric analysis of TH+ striatal innervation. E) HPLC characterization of striatal dopamine content. F) Open field behavioral analysis displaying percent time spent in the center of the open field arena. G) Representative heat map images of open field behavior motion tracking indicating movement and latency within the arena. (Statistics: All *p<0.05, **p<0.01, ****p<0.0001).

We next assessed the level of nigrostriatal protection that intrabody reduction of pathology could yield. Stereological estimates of TH+ neurons in the SN showed the lesion paradigm was able to create a substantial deficit in dopaminergic neurons in the SN (Fig. 5a,b) and TH+ striatal terminals (Fig. 5c,d) compared to controls (Control: 20073±2936 DA neurons, Lesion: 8816±4407 DA neurons, p<0.0001). We observed significant preservation of dopaminergic neurons in animals treated with VH14-hPEST (16612±3078 DA neurons, p<0.01), but not with VH14-P426A (12709±3212 DA neurons) (Fig. 5b). Densitometric analysis of TH+ striatal terminals also verified VH14-hPEST was the only intrabody capable of preserving striatal innervation (Fig. 5d). We then assessed the corresponding levels of striatal dopamine by HPLC which confirmed VH14-hPEST delivered superior rescue in comparison to VH14-P426A (Fig. 5e).

To establish whether structural nigrostriatal preservation yielded any effects on functional phenotypes, we analyzed open field behavior at 19-weeks post-treatment (Fig. 5f, Fig.S3). Lesioned animals showed significant aversiveness to arena center (an indicator of anxiety-like behavior) compared to control animals, which was corrected in animals treated with VH14-hPEST (p<0.05, Fig. 5f,g). Other open field behaviors, such as velocity (Fig.S4f) and total locomotor behavior (Fig.S4g) showed identical trends from VH14-hPEST treatment, but were not statistically significant due to high performance variability. We also conducted narrow beam analysis to assess gait disturbances (Fig.S4j,k). Both task initiation (Fig.S4j) and latency to goal (Fig.S4k) in VH14-PTAP-treated animals were non-significant due to high variability (Fig.S4j,k). These data verify intranigral injections of both VH14-hPEST and VH14-P426A can mitigate the development of nigrostriatal αSyn pathology, but VH14-hPEST shows evidence of enhanced neuroanatomic and functional rescue from pathological perturbations.

### Global CNS delivery of AAV-PTAP VH14 intrabodies through the cisterna magna provides sufficient nigrostriatal targeting and prevents the propagation of αSyn pathology across the neuraxis

**Figure 6.**
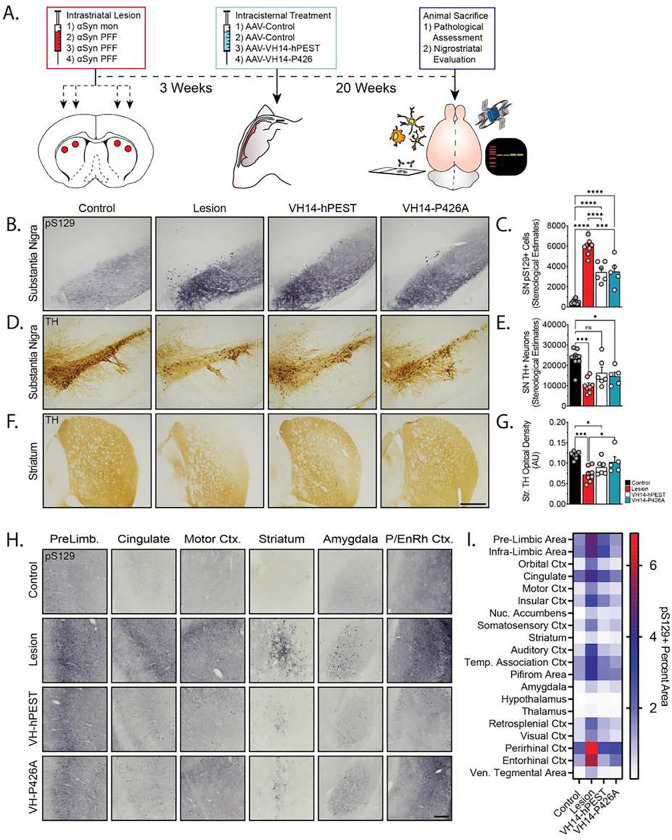
CSF infusions of VH14-PTAP intrabodies via the cisterna magna provide widespread protection from pathological progression. A) Study design: Rats received bilateral dual lesion injections of αSyn PFFs, or Syn monomers for controls, into the striatum. Three weeks post-lesion, animals received intracisternal injections of AAV carrying VH14-hPEST, VH14-P426A, or B8-hPEST control. Animals were sacrificed after 20-weeks post-treatment. B) Representative microphotographs of pS129+ stained αSyn pathology in the SN and C) corresponding stereological estimations of pS129+ aggregates. D) Images of microphotographs of TH-ir dopaminergic neurons in the SN and E) corresponding stereological estimates of SN TH+ neurons. F) Representative images of striatal terminals labeled with TH and G) densitometric analysis of TH+ striatal innervation. H) Brightfield images of pS129+ staining in selected regions of interest vulnerable to αSyn pathology in striatal PFF model. I) Heat map of quantified percent area stained with pS129 demonstrating reductions of pathology in VH14-PTAP treated cohorts. Mean values are displayed on heat map. (Statistics: All *p<0.05, ***p<0.001, ****p<0.0001).

Advanced Lewy pathology is evident throughout the neuraxis of PD patients and patients with other clinical synucleinopathies. Thus, an important potential therapeutic application for intrabody targeting of αSyn is the ability to provide widespread neuroprotection from pathological αSyn. We determined whether global CNS distribution of VH14-PTAP constructs would hinder the pathological spread and seeding to brain regions vulnerable to pathologic αSyn deposition. We have previously characterized the widespread transduction efficiency of the engineered capsid, AAV.PHP.eB, when delivered into the CSF via the cisterna magna of older rats^40^. Thus, we aimed to utilize this delivery platform to target cell populations naïve to αSyn synuclein insult. To induce pathological progression and seeding, we bilaterally injected αSyn PFFs (Fig.S1B), or αSyn monomers for control subjects, into two sites of the striata of adult rats, as previously described (Fig. 6a)^39^. Three weeks after induction of pathology, we intracisternally infused viral vectors carrying the B8-hPEST intrabody for control subjects, and either B8-hPEST, VH14-hPEST or VH14-P426A into PFF or monomer animals (Fig. 6a). Following 20 weeks, animals were sacrificed for histopathological assessments (Fig. 6a).

We first assessed whether CSF-transport of intrabody vectors could provide adequate transduction of the SN to engage the nigrostriatal deposition of αSyn (Fig. 6b,c). Indeed, our previous characterization of AAV.PHP.eB biodistribution verified increased levels of nigral transduction compared to its parent serotype, AAV9^40^. As expected, stereological estimates of pS129+ aggregates in lesioned animals (5912±774 pS129+ aggregates) showed significant levels of pathology and negligible aggregate counts in animals injected with monomeric αSyn (518±212 pS129+ aggregates) (p<0.0001, Fig. 6c). However, we observed statistically significant reductions of pS129+ labeled pathology in animals treated with VH14-hPEST (3448±948 pS129+ aggregates, p<0.0001) (Fig. 6c). VH14-P426A also demonstrated a statistically significant reduction of pathology compared to B8-hPEST-treated, lesioned animals (3492±1356 pS129+ aggregates, p<0.001) (Fig. 6c). Again, next we analyzed nigrostriatal dopaminergic integrity with stereological assessments of nigral TH+ neurons (Fig. 6d,e) and densitometric analysis of TH+ striatal terminals (Fig. 6f,g). Corresponding with the pS129 pathological assessment, we observed a statistically significant rescue of striatal TH optical density by VH14-P426A (p<0.05, Fig. 6f,g), but not a significant protection against nigral DA neurons loss (Fig. 6e). We did not observe a statistically significant protection against DA neuron loss in either VH14-PTAP-treated cohorts.

To evaluate whether global CNS transduction of PTAP intrabodies could protect regional populations of neurons from impending pathology, we assessed the level of pS129+ immunoreactivity throughout regions vulnerable to αSyn aggregation in this model system (Fig. 6h). As anticipated, negligible pS129+ signal was observed across brain regions in animals injected with αSyn monomers (Fig. 6h). B8-hPEST-treated animals that received intrastriatal PFF injections showed substantial pathology throughout both cortical and subcortical brain regions, with robust aggregate formation particularly at the prelimbic area, cingulate gyrus, motor cortex, striatum, amygdala, and perirhinal and entorhinal cortex (Fig. 6h). In both intrabody treated cohorts, we observed graded decreases of pS129+ soma and neuritic staining across every region we assessed, including the immediate periphery of the site of injection (Fig. 6h). To quantify decreases in pathology, we determined the percent area stained pS129+ for each region of interest (Fig.S5) and accumulated the data in a heat map (average % pS129+) to show pathological discrepancies (Fig. 6i). Both intrabody treatments demonstrated decreased pathology across each region, with VH14-P426-treated animals demonstrating more significant reductions, predominantly in rostral brain regions such as pre-/infralimbic areas and the cingulate cortex (Fig. 6i). Importantly, these data provide contextual evidence that intracellular anti-aggregation or αSyn-regulating strategies may provide neuroprotection from the extracellular cell-to-cell propagation of pathological αSyn.

## Discussion

**Figure 7.**
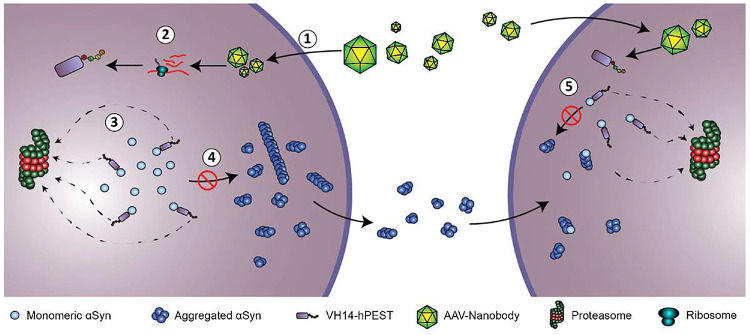
Model of global CNS VH14-hPEST mediated mode of action in slowing αSyn pathophysiology. 1) AAVs carrying intrabody plasmids transduce target cells and 2) constitutively generate VH14-hPEST intracellularly. 3) VH14-hPEST reduces intracellular αSyn levels via proteasomal degradation and interferes with binding epitopes permissive to seeding and templating, 4) thus, preventing the formation of aggregates to be released into the extracellular space. 5) AAV-VH14-hPEST is transduced in neurons naïve to pathological aggregation. 5) VH14-hPEST reduces available αSyn substrate for proteopathic seeds that may propagate from dysfunctional cells and tempers seeding and aggregation.

Collectively, these data point towards the neuroprotective potential of αSyn-targeting intrabodies, with differential effects of therapy contingent on the degradation capacity of PEST fusions. Based on our data, we propose an updated mechanistic model for the action of αSyn-targeting intrabodies (Fig. 7). We determined that viral vector-mediated delivery of VH14-PTAP intrabodies to cells susceptible to αSyn aggregation can inhibit the rate of pathological deposition and hinder the toxic actions of said pathology which may occur through two primary mechanisms: 1) the promotion of αSyn turnover and quieting of proteostatic stress, and 2) the direct interference of αSyn aggregate binding motifs. Importantly, our data are consistent with the hypothesis that viral delivery of intrabodies can facilitate protection through the reduction in availability of substrate for seeding and aggregate maturity in naïve cells that may act as recipient populations for propagating proteopathic αSyn seeds (Fig. 7). Thus, intrabody-based αSyn therapeutics may provide significant intracellular protection against αSyn toxicity as well as mitigate the cell-to-cell transfer of αSyn pathology.

Although evidence points toward extracellular transmission of αSyn and Lewy pathology as contributors to pathophysiology in clinical synucleinopathies^41–43^, αSyn aggregate formation can initiate as stochastic, intracellular events that do not require donor proteopathic seeds to induce templating. Recent results in clinical trials for immunotherapeutic modalities targeting extracellular αSyn have been discouraging^44^, and may indicate inadequate target selectivity, ineffective target engagement, or an inadequate supply of extracellular αSyn proteoforms to bind to. Indeed, the exact mode of αSyn transmission and latency of αSyn proteoforms in the extracellular space is still unknown in the context of clinical PD^43,45^, drawing question to how amenable pathological αSyn in the extracellular space is to immunotherapeutic intervention strategies that target only this space. However, it is important to note that intrabody therapeutics may be used in combinatorial approaches to also target extracellular species, via use of monoclonal antibodies or fusion antibody fragments^46,47^, or through AAV vectors that include genes encoding both intracellular and secreted engineered antibody fragments.

Our primary objective in this study was to utilize a cell-autonomous therapeutic approach in the form of intrabodies. To that end, we utilized a fully human construct that qualifies for further pre-clinical testing. We have previously shown that the human VH14 binder fused to a murine PEST degron is capable of ameliorating αSyn-induced neurotoxicity when administered to an intranigral αSyn overexpression model^26^. Here, we validated that the fusion of a PEST sequence of human origin to VH14 can achieve equivalent levels of αSyn turnover. Importantly, we also validated that the fully human VH14-hPEST intrabody can effectively engage αSyn in a disease-relevant, human-cell based system with respect to 3X-*SNCA* 3D organoids. These are the first experiments illustrating VH14 intrabody target engagement in human cell populations, which is informative in mitigating the translational risk of therapeutic constructs.

The results reported here are of particular interest as they feature a method to program the levels of reduction of the intracellular αSyn. The definitive function of αSyn in neurons remains undetermined^48^, although several studies have indicated a role for αSyn in binding lipid membranes^49–51^ and mediating vesicular transmission at the synaptic terminal^50,52–54^. However, the functional requirement of αSyn protein in the brain is conflicting. *SNCA*^−/−^ mice display neither outright neurodegeneration nor major alterations in striatal neurotransmission^55^. αSyn null mice also show enhanced dopaminergic transmission and protection from the mitochondrial neurotoxin, MPTP ^56^. Studies have also shown that knockdown of αSyn levels with interfering RNA shows no deleterious effects in rats for up to a year^57,58^. These findings are in direct contrast with two reports showing dose-dependent nigral neuroinflammation and subsequent neurodegeneration upon shRNA-mediated αSyn knockdown^59,60^ and these conflicting data may be due to the degree of αSyn knockdown. Thus, although an optimal physiological level for neuronal αSyn is unclear, the targeting of intracellular αSyn remains a viable and attractive therapeutic path. Antisense oligonucleotide (ASO) technologies have demonstrated pre-clinical success in suppressing αSyn expression and protecting monoamine (primarily dopaminergic) neurons^61–64^, although this approach does not offer readily programmable levels of suppression or feature direct engagement with target protien.

Additionally, the creation of PTAP intrabodies against αSyn may now be utilized as tools for nuanced understanding of the αSyn expression level necessity in different cell populations^65^. We observed successful control over intracellular αSyn levels in ST14A cells, with variants ranging from 30–60% of control αSyn expression. Interestingly, in the current study we observed differential phenotypic rescue and efficacy by PTAP intrabodies depending on the model system we used and the method of treatment delivery. The parent VH14-hPEST intrabody provided the greatest nigrostriatal preservation against the dual intranigral lesion model compared to the VH14-P426A degron variant, which was the strongest degrader (~60%) in our *in vitro* screen. Interestingly, protection of nigrostriatal TH immunoreactivity by VH14-hPEST did not correspond with the more significant reductions in nigral pS129+ pathology observed after treatment with VH14-P426A. However, the intracisternal delivery and transduction of AAV-VH14-P426A enabled superior nigrostriatal protection and mitigation of pathological deposition across most of the brain regions we analyzed. It is unknown whether the reductions across global CNS pathologies in VH14-PTAP-treated cohorts is the result of direct intrabody action on transduced target cell populations or the gating of local and transsynaptic pathological spread upstream of proteopathic transmission to vulnerable network regions.

To contextualize these findings, it may be an effective strategy to employ more potent anti-αSyn intrabodies (VH14-P426A) in situations in which delivery mechanisms may not provide sufficient coverage or transduction efficiency. However, in the case of direct delivery strategies, such as the intranigral paradigm we employed in this report, lower-efficiency degrading constructs (VH14-hPEST) may provide more effective neuroprotection in more concentrated target regions. There is a tremendous amount of research in progress to develop AAV variants that can transduce the brain more efficiently^66^ which we hope to employ in future studies.

There were several findings from this study that warrant future investigation. First, it is noteworthy that the predicted efficiency of proteasomal turnover for VH14-PTAP intrabodies decreased with each layer of complexity added to the model system. In 3D-organoid cultures and *in vivo* experiments, we did not observe the near ~60% ablation of αSyn signal achieved in the 2D cell culture screens with VH14-P426A. Immunoblot data from *in vivo* targeting in intranigral lesion animals also only showed modest decreases in αSyn signal in VH14-P426A-treated animals, with minimal disparities in the degradation efficiency between VH14-P426A and VH14-hPEST. The dampening effect may have been the result of inconsistencies in intrabody expression systems where viral vector delivery may not transduce cells or express intrabodies as efficiently as the PEI-transfection systems utilized in our 2D culture. Varying multiplicity of infection (MOI) may confound readouts of true target engagement, and proteasomal turnover efficiency, of intrabody constructs. It may also be possible that noise from off-target or peripheral cell populations in 3D-organoids and animal tissue may lower the resolution of detectable αSyn expression changes. Immunoblots from our *in vivo* investigations were generated from midbrain lysates in which αSyn content from extranigral cells contribute to overall total αSyn signal. Further investigation using techniques with single-cell resolution may be necessary to understand PTAP regulation of αSyn within specific individual transduced cells. These will be critical to understand the effects of αSyn titration on cellular function and proteopathic potential.

An additional consideration to address in future studies will be the question of αSyn thresholds for neuronal function. In our *in vivo* efficacy studies, we observed no outright deficit or exacerbation of toxicity and neurodegeneration in either intrabody-treated cohort compared to B8-treated lesioned animals, thus signaling intrabody use at these concentrations do not induce perceptible αSyn loss-of-function injury. Although we observed robust nigral pathology and resultant loss of nigrostriatal dopaminergic tone, we only observed minor functional deficits in lesioned animals due to largely variable animal performance in behavioral tasks, obscuring a clean readout for intrabody-mediated functional rescue. Bilateral lesions, as were performed in these studies, variably induce movement deficits which most gross motor assays may not be sensitive enough to detect. Indeed, two recent studies using PFFs to induce synucleinopathy have also observed a lack of robust motor phenotype or no outright balance dysfunction or akinesia^65,67^, drawing attention to the considerable functional variability that exists when employing these animal model systems. Future studies should incorporate the investigation of the long-term intrabody action in living neurons, including how programming intracellular αSyn content may affect electrophysiological properties of treated cells, especially the dopaminergic neuron populations. For our proof-of-concept *in vivo* study, we used retired breeder female rats to provide a middle-aged cohort of animals for these experiments. For full pre-clinical testing, older male rat cohorts will also be incorporated to account for any sex differences in αSyn mediation.

Together, this series of *in vivo* and *in situ* studies show the promising potential of intracellular targeting of αSyn with intrabody therapy. Fully human PTAP variants fused to a human anti-αSyn nanobody constructs provide programmable tools for therapeutic protein modulation and characterization, and may be an effective strategy to employ with other nanobody reagents in the future.

## Materials and Methods

### Animals

Animal studies were approved by the Institutional Animal Care and Use Committee at Rush University Medical Center. Sixty-four, 7–10-month-old, female Sprague-Dawley rats (Charles River) were maintained under a 12-hour light-dark cycle at 25°C and 50% humidity. Animals were distributed randomly amongst lesion and treatment injection groups, correcting for body weight. Experimenters were blinded to animal lesion and treatment cohorts for all in-life behavioral analysis.

### Cell Culture and Transfection

Undifferentiated ST14A cells, a rat neuronal cell line, or Human Embryonic Kidney (HEK) 293 were cultured and transfected using PEI with intrabody constructs as previously described^27^. Briefly, cells were transfected with 2μg of DNA across a 6-well plate using JetPEI transfection reagent (Polyplus Transfection Inc) and imaged and harvested 72-hours post-transfection. Cells were transfected with empty vector control plasmids or engineered VH14-PTAP intrabody constructs.

### Generating 3D forebrain organoids derived from human iPSCs

Human iPSCs (Cell Line ND3491)^33^ were cultured on Matrigel matrix (Corning #356231) coated 6-well plates (Corning #3506) with mTeSR (StemCell Technologies #85851). Cultures were expanded and split every 5–7 days when they reached ~70–80% confluency in ReLeSR (StemCell Technologies #5873). When generating spheroids, iPSCs were rinsed with DMEM/F12 (Thermo Fisher #11330–032) then dissociated into single cell suspension with Accutase (Thermo Fisher #A1110501) and incubated at 37°C in 95% O_2_/5% CO_2_ incubator for 5 min or until cells detached. The cells were then transferred cells to a 15 mL canonical tube and counted on cytoSMART Corning Cell Counter. The cell suspension was centrifuged at 1,200 rpm for 4 min and resuspended at 10,000 cells per 100 ul or 1E6 cells per 10 mL in mTeSR medium supplemented with 10 μM rock inhibitor Y-27632 (Tocris #1254). The resuspended cells were transferred to a small trough and with a multi-channel, gently pipetted, and evenly distributed 100 ul per well into the 96 slit well plate (Prime Surface #MS9096SZ). The plate was centrifuged at 100 xrcf for 3 min. The organoids received daily half feeds for five days with E6 medium supplemented with 2.5 μM Dorsomorphin (DM), 10 μM SB4311541, and 2.5 μM XAV-939 (Tocris #XAV-939). On day 6, the spheroids were transferred into Neuralbasal A (ThermoFisher #10888022) with B27 (without A, 10888022), 1% anti-A, 1% GlutaMAX, 20 ng/mL FGF2 (Shenandoah #100–28) and 20 ng/mL EGF (Shenandoah #100–26) for daily for 10 days, and every other day for following 9 days. From day 43, we fed with the same medium every 4 days without growth factors. Forebrain organoids were transduced with lentivirus 2-months after prep date. Organoids were collected at 4-months for immunostaining, western blot lysates and RNA sequencing.

### Intrabody Construct and Plasmid Design

VH14-hPEST and respective hPEST variants were cloned into pcDNA3.1 (+), pAAV-MCS, and pTetO-FUW expression plasmids. To identify expression of the intrabodies, a hemagglutinin (HA) epitope tag (amino acid sequence YPYDVPDYA) was fused to the C-terminal end of the intrabodies^27,28^. To direct the intrabodies and their cargo to the proteasome, a standard PEST motif corresponding to amino acids 422–461 (NPDFPPEVEEQDASTLPVSCAWESGMKRHRAACASASINV) from human ODC (GenBank accession number AH002917.2) was added C-terminal end of the HA-tag. To alter the targeted degradation of the intrabody and its bound antigen, single (D433A, C441A, S445A) and compound (P426A/P427A) mutations within the hPEST degron were synthesized commercially (GenScript, USA). To control for the effects of both protein overexpression and proteasome degradation of PEST-tagged intrabodies, we generated a control single domain intrabody against an antigen not expressed in any of our test systems. VHH-B8 is a well characterized camelid nanobody that binds to Botulinum Neurotoxin^29^ and has demonstrated excellent intracellular solubility in our test system^5^. The intrabodies were subcloned with standard cloning techniques into pcDNA3.1^−^and pAAV-MCS according to the following cloning strategy: XbaI-intrabody-NotI-HA-PEST degron-HindIII. All expression plasmids were verified by Sanger DNA sequencing (Genewiz, NJ) and prepared with Nucleobind Xtra Midi Endotoxin free (Takara #740420.5) prep kits according to manufacturer's protocol.

### Lentivirus vector production and viral packaging

VH14 PTAP intrabodies were cloned into an inducible tetracycline on 3rd generation lentiviral vector, pTet-O-Ngn2-puro, (gift from Marius Wernig; Addgene plasmid # 52047)^68^. The Ngn2 cassette was replaced with VH14-hPEST and respective hPEST variants at the 5’ EcoRI and 3’ Xba1 cloning sites. The tetracycline on system is reverse tetracycline-controlled transactivator, FUW-M2rtTA (rtTA) dependent (gift from Rudolf Jaenisch, Addgene plasmid # 20342)^69^. Lentiviruses were produced in 293FT cells (ThermoFisher; Cat# R70007). The intrabody expressing pTet-O-puro or rtTA plasmids (5.6 μg) were co-transfected with plasmids pVSV-G (7.1 μg) and pCMVd8.9 (14.2 μg) into 293FT cells with 122 μg of transfection grade linear polyethylenimine hydrochloride (MW 40,000) (PEI MAX; Polyscience Inc; Cat# 24765) solution. Lentiviral envelope and packaging plasmids pVSV-G and pCMVd8.9 were a gift from Sally Temple^70^. Transfection medium was changed after 3 hrs. Viral-containing media was collected at 48 and 72 hrs in sterile 50 mL conical tube, and centrifuged at 500 RCF for 10 min. The viral supernatant was then filtered through a 0.45 μm cellulose acetate low protein binding filter (Corning, Cat# 09–761-35) and stored at 4°C for ~2–24 hrs respectively before proceeding to next step. Viral supernatants were concentrated in Lenti-X Concentrator (Takara Bio; Cat# 631232) according to manufacturer’s protocol and resuspended in 1X CMF PBS. Lentivirus titers were determined by qRT-PCR lentivirus titration kit (ABM, Cat# LV900). For viral transduction, lentiviral vectors at a multiplicity of infection (MOI) of 1 were added to organoids.

### AAV Design and Prep for In Vivo Studies

Anti-αSyn and control intrabodies were cloned into pAAV-MCS. Ampicillin resistant plasmids were transformed into electrocompetent SURE2 cells (Agilent Technologies, Santa Clara, CA, USA), inoculated into 1L LB + Amp and shaken at 220rpm at 30°C for 12–16 hours. Bacteria was harvested by 20 min centrifugation at 4,00 rpm. Plasmid DNA was isolated using an Invitrogen^™^ PURELINK^™^ HiPure Plasmid Maxiprep Kit. Following plasmid isolation, further purification as achieved following Phenol:Chloroform:Isoamyl Alcohol extraction. Genomes were packaged into AAV-PHP.eB using polyethylenimine (PEI) triple transfection into adherent HEK293T cells. Cells and media were harvested 72 hours post-transfection, and viral particles were collected from media using polyethylene glycol precipitation (PEGylation). Vector purification was performed using an OptiPrep^™^ Iodixanol Density Gradient Medium (Sigma-Aldrich, St. Louis, MO, USA). Viral titers were assessed using Bio-Rad QX 200 Droplet Digital PCR system, droplet generator, plate sealer, and thermal cycler (Bio-Rad, Hercules, CA, USA), and diluted to 1×10^12^ vector genomes (vg)/mL using balanced salt solution^71,72^.

### Behavioral Analysis

#### Open Field

Total locomotor activity and anxiety-related behavior were tested in an open field arena (40×40 cm, 30 cm wall height) and were recorded for Noldus behavioral analysis (EthoVisionXT). End stage animals were introduced to the center of the apparatus for 300 second intervals. Arena was wiped down with ethanol between each trial to prevent olfaction-based investigation. Locomotor activity was determined by automated tracking software monitoring the distance traveled and velocity of the designated center of the animal. Anxiety-like behavior was determined as a ratio of animal presence in the 15×15 cm center box of the arena vs the periphery. Additional parameters included the ratio of movement time to non-movement time.

### Narrow Beam

Narrow beam test was performed to measure balance and kinetic performance. Animals were placed in reverse orientation and were allowed to traverse an elevated wooden beam spanning 100 cm to and darkened end goal box they were familiarized with prior to testing. The time to initiate task, foot slips, and latency to goal box entry were recorded. A maximum of 300 second timecourse was permitted before animals were recorded to be non-compliant. Animals were tested at baseline and re-tested at two-month intervals until the conclusion of the study.

### Stereotactic Surgery for Animal Lesion and Treatment

Stereotaxic surgical procedures were performed in accordance to protocols approved by the Rush University Medical Center IACUC. Rats were evenly divided into two primary study cohorts (32 rats each). Animals were deeply anesthetized with sodium ketamine hydrochloride (concentrations) and immobilized in a stereotaxic frame. Intranigral lesions were delivered with an adapted protocol from the previously described report^36^. Briefly, animals were bilaterally injected with two combined infusions (3μL) of AAV6-αSyn (8.8×10^8^ vg/ml) and sonicated PFFs (10μg) at the following coordinates: AP=−5.3, ML=±1.6, DV=−7.2 and AP=−5.3, ML=±2.6, DV=−6.7. For all control injections, equal concentrations of αSyn monomer and equititer AAV6-GFP were injected. Injections were made at a flow rate of 1 uL/min with a 4 minute dwell-period following injection. Three-weeks post lesion, animals underwent a second round of surgery using identical injection targets for AAV intrabody treatment. Animals received treatment four 2μL injections of AAV.PHP.eB expressing B8-hPEST, VH14-hPEST, or VH14-P426A.

A second cohort of experimental animals were used to determine the effects of global CNS delivery of intrabody constructs. The intrastriatal lesion paradigm utilized for these animals followed protocols previously described^39^. Following three weeks post-model induction, animals received 50uL infusions of AAV.PHP.eB expressing B8-hPEST, VH14-hPEST, or VH14-P426A into the cisterna magna, following protocols described in Chatterjee, et. al. 2022^40^.

### Tissue Processing

Animals at study endpoint were run through end-stage behavioral testing and were deeply anesthetized with ketamine/xylazine. Animals were perfused transcardially with cold 0.9% phosphate-buffered saline (PBS) solution, hemisected, and divided with one hemisphere being immersion-fixed in 4% paraformaldehyde solution overnight at 4°C and the alternate hemisphere being dissected for molecular processing. Fixed brains were transferred through a sucrose gradient until sunken and were cut on a frozen-stage sliding microtome at 40μm intervals. Sections were stored in cryopreservative buffer until histological processing. Frozen tissue regions were pulverized under liquid nitrogen and fractioned into aliquots for molecular processing.

### Immunohistochemistry and Fluorescent Labeling

All immunohistochemistry and immunofluorescent labeling strategies were adapted from protocols as previously described^26^. Briefly, for DAB labeling, free-floating sections were quenched with sodium periodate, blocked, and incubated with primary antibodies (Table 1). Sections were labeled with biotinylated secondary antibodies (Table 1) and conjugated with HRP Biotin/Avidin Complex ABC kit (Vector Laboratories). DAB stain was developed with hydrogen peroxide and sections were mounted, dehydrated through graded alcohols, and coverslipped. Immunofluorescent labeling followed identical protocols without the DAB step. Sections were mounted and coverslipped with DPX mounting resin.

### Stereology, Densitometric Analysis, and Quantification of Pathology

Stereological estimates of TH+ neurons and pS129+ aggregates were performed by unbiased counting methods using the optical fractionator probe in StereoInvestigator (MBF Biosciences), as previously described. Stereological parameters for each target are listed in Table 2. Optical density measurements were made on images acquired by an inverted Nikon A1R confocal microscope using ImageJ software (NIH version 1.52a). Contoured regions were traced from four consecutive, level-matched sections and measured for mean fluorescent signal. For regional pathological analysis of pS129+ staining, tiled 10X microphotographs of level-matched sections were acquired using equivalent acquisition settings. Images were converted to grayscale (16-bit) and staining was identified using thresholding to develop a binary mask of pathology. Regions of interest were outlined in accordance with neuroanatomic landmarks as described in the Allen Brain Atlas (Fig.S5). The area stained was recorded with respect to total outlined regional area.

### Immunoblotting

Frozen midbrain tissue or cell pelets was homogenized in a standard RIPA buffer with protease and phosphatase inhibitors and the supernatant extracted after high-speed centrifugation. Protein concentrations were determined using a BCA assay compared to protein standards of bovine serum albumin. Samples were normalized for protein concentration and reduced with BME. Twenty ug of protein were resolved on 4–12% gradient bis-tris SDS polyacrylamide gels and transferred onto 0.4 um nitrocellulose membranes. Membranes were fixed with 0.4% paraformaldehyde for 20 minutes and washed with water for 10 minutes prior to blocking. Blots were blocked overnight in Odyssey Blocking Buffer and probed for two hours with primary antibodies listed in table 1. Blots were exposed to corresponding secondary antibodies (table 1) for one hour, washed away, and stored under PBS until image acquisition. Images were scanned on an Odyssey near-IR scanning module.

### HPLC

High-performance liquid chromatography protocols were performed as previously described. Briefly, striatal tissue from animals was carefully dissected from forebrain tissue and homogenized with 0.2M perchloric acid solvated in isoproterenol. Striatal homogenates were centrifuged at 16000 rpm and the supernatant was extracted. Ten uL samples of supernatant was injected into a EIcompak SC-3ODS column and compared to a standard curve to analyze concentrations of dopamine. All samples were analyzed in accordance with manufacturer’s protocol.

### Statistical Analysis

All statistical analysis was performed using GraphPad Prism software (GraphPad, Version 9.02). With the exception of data sets with multiple time points, all data were subject to normality testing and analyzed via one-way ANOVA with a Tukey post-hoc test for multiple comparisons. Timecourse data was analyzed using a two-factor ANOVA (treatment and time) with Sidak’s post-hoc test for multiple comparisons. Individual statistical parameters that deviate from protocols are included in figure legends. All featured data is represented as Mean ± SEM with *p<0.05, **p<0.01, ***p<0.001, and ****p<0.0001.

## References

[1] Goedert M., Jakes R. & Spillantini M. G. The Synucleinopathies: Twenty Years On. J Parkinsons Dis 7, S51–S69, doi:10.3233/JPD-179005 (2017). 28282814 PMC5345650

[2] Chartier-Harlin M. C. Alpha-synuclein locus duplication as a cause of familial Parkinson's disease. Lancet 364, 1167–1169, doi:10.1016/S0140-6736(04)17103-1 (2004). 15451224

[3] Ibanez P. Causal relation between alpha-synuclein gene duplication and familial Parkinson's disease. Lancet 364, 1169–1171, doi:10.1016/S0140-6736(04)17104-3 (2004). 15451225

[4] Singleton A. B. alpha-Synuclein locus triplication causes Parkinson's disease. Science 302, 841, doi:10.1126/science.1090278 (2003). 14593171

[5] Appel-Cresswell S. Alpha-synuclein p.H50Q, a novel pathogenic mutation for Parkinson's disease. Mov Disord 28, 811–813, doi:10.1002/mds.25421 (2013). 23457019

[6] Lesage S. G51D alpha-synuclein mutation causes a novel parkinsonian-pyramidal syndrome. Ann Neurol 73, 459–471, doi:10.1002/ana.23894 (2013). 23526723

[7] Liu H. A Novel SNCA A30G Mutation Causes Familial Parkinson's Disease. Mov Disord 36, 1624–1633, doi:10.1002/mds.28534 (2021). 33617693

[8] Pasanen P. Novel alpha-synuclein mutation A53E associated with atypical multiple system atrophy and Parkinson's disease-type pathology. Neurobiol Aging 35, 2180 e2181–2185, doi:10.1016/j.neurobiolaging.2014.03.024 (2014). 24746362

[9] Polymeropoulos M. H. Mutation in the alpha-synuclein gene identified in families with Parkinson's disease. Science 276, 2045–2047, doi:10.1126/science.276.5321.2045 (1997). 9197268

[10] Proukakis C. A novel alpha-synuclein missense mutation in Parkinson disease. Neurology 80, 1062–1064, doi:10.1212/WNL.0b013e31828727ba (2013). 23427326 PMC3653201

[11] Zarranz J. J. The new mutation, E46K, of alpha-synuclein causes Parkinson and Lewy body dementia. Ann Neurol 55, 164–173, doi:10.1002/ana.10795 (2004). 14755719

[12] Chia R. Genome sequencing analysis identifies new loci associated with Lewy body dementia and provides insights into its genetic architecture. Nat Genet 53, 294–303, doi:10.1038/s41588-021-00785-3 (2021). 33589841 PMC7946812

[13] Du T. T. GBA deficiency promotes SNCA/alpha-synuclein accumulation through autophagic inhibition by inactivated PPP2A. Autophagy 11, 1803–1820, doi:10.1080/15548627.2015.1086055 (2015). 26378614 PMC4824589

[14] Moors T. E. Therapeutic potential of autophagy-enhancing agents in Parkinson's disease. Mol Neurodegener 12, 11, doi:10.1186/s13024-017-0154-3 (2017). 28122627 PMC5267440

[15] Stefanis L. How is alpha-synuclein cleared from the cell? J Neurochem 150, 577–590, doi:10.1111/jnc.14704 (2019). 31069800

[16] Spillantini M. G. Alpha-synuclein in Lewy bodies. Nature 388, 839–840, doi:10.1038/42166 (1997). 9278044

[17] Spillantini M. G. Filamentous alpha-synuclein inclusions link multiple system atrophy with Parkinson's disease and dementia with Lewy bodies. Neurosci Lett 251, 205–208, doi:10.1016/s0304-3940(98)00504-7 (1998). 9726379

[18] Wong Y. C. & Krainc D. alpha-synuclein toxicity in neurodegeneration: mechanism and therapeutic strategies. Nat Med 23, 1–13, doi:10.1038/nm.4269 (2017). PMC8480197 28170377

[19] Menon S. Alpha-Synuclein Targeting Therapeutics for Parkinson's Disease and Related Synucleinopathies. Front Neurol 13, 852003, doi:10.3389/fneur.2022.852003 (2022). 35614915 PMC9124903

[20] Rodger A. T., M A. L. & Carter W. G. Are Therapies That Target alpha-Synuclein Effective at Halting Parkinson's Disease Progression? A Systematic Review. Int J Mol Sci 24, doi:10.3390/ijms241311022 (2023). PMC10341763 37446200

[21] Messer A. & Butler D. C. Optimizing intracellular antibodies (intrabodies/nanobodies) to treat neurodegenerative disorders. Neurobiol Dis 134, 104619, doi:10.1016/j.nbd.2019.104619 (2020). 31669671

[22] Lynch S. M., Zhou C. & Messer A. An scFv intrabody against the nonamyloid component of alpha-synuclein reduces intracellular aggregation and toxicity. J Mol Biol 377, 136–147, doi:10.1016/j.jmb.2007.11.096 (2008). 18237741 PMC2359154

[23] Chen Y. H. Downregulation of alpha-Synuclein Protein Levels by an Intracellular Single-Chain Antibody. J Parkinsons Dis 10, 573–590, doi:10.3233/JPD-191787 (2020). 32176654

[24] Chen Y. H. Administration of AAV-Alpha Synuclein NAC Antibody Improves Locomotor Behavior in Rats Overexpressing Alpha Synuclein. Genes (Basel) 12, doi:10.3390/genes12060948 (2021). PMC8233769 34205689

[25] Asaadi Y., Jouneghani F. F., Janani S. & Rahbarizadeh F. A comprehensive comparison between camelid nanobodies and single chain variable fragments. Biomark Res 9, 87, doi:10.1186/s40364-021-00332-6 (2021). 34863296 PMC8642758

[26] Chatterjee D. Proteasome-targeted nanobodies alleviate pathology and functional decline in an alpha-synuclein-based Parkinson's disease model. NPJ Parkinsons Dis 4, 25, doi:10.1038/s41531-018-0062-4 (2018). 30155513 PMC6105584

[27] Butler D. C. Bifunctional Anti-Non-Amyloid Component alpha-Synuclein Nanobodies Are Protective In Situ. PLoS One 11, e0165964, doi:10.1371/journal.pone.0165964 (2016). 27824888 PMC5100967

[28] Joshi S. N., Butler D. C. & Messer A. Fusion to a highly charged proteasomal retargeting sequence increases soluble cytoplasmic expression and efficacy of diverse anti-synuclein intrabodies. MAbs 4, 686–693, doi:10.4161/mabs.21696 (2012). 22929188 PMC3502235

[29] Tremblay J. M. Camelid single domain antibodies (VHHs) as neuronal cell intrabody binding agents and inhibitors of Clostridium botulinum neurotoxin (BoNT) proteases. Toxicon 56, 990–998, doi:10.1016/j.toxicon.2010.07.003 (2010). 20637220 PMC2946066

[30] Ghoda L., van Daalen Wetters T., Macrae M., Ascherman D. & Coffino P. Prevention of rapid intracellular degradation of ODC by a carboxyl-terminal truncation. Science 243, 1493–1495, doi:10.1126/science.2928784 (1989). 2928784

[31] Takeuchi J., Chen H. & Coffino P. Proteasome substrate degradation requires association plus extended peptide. EMBO J 26, 123–131, doi:10.1038/sj.emboj.7601476 (2007). 17170706 PMC1782366

[32] Zhang M., Pickart C. M. & Coffino P. Determinants of proteasome recognition of ornithine decarboxylase, a ubiquitin-independent substrate. EMBO J 22, 1488–1496, doi:10.1093/emboj/cdg158 (2003). 12660156 PMC152902

[33] Lin L. Molecular Features Underlying Neurodegeneration Identified through In Vitro Modeling of Genetically Diverse Parkinson's Disease Patients. Cell Rep 15, 2411–2426, doi:10.1016/j.celrep.2016.05.022 (2016). 27264186

[34] Bowles K. R. ELAVL4, splicing, and glutamatergic dysfunction precede neuron loss in MAPT mutation cerebral organoids. Cell 184, 4547–4563 e4517, doi:10.1016/j.cell.2021.07.003 (2021). 34314701 PMC8635409

[35] Negrini M. Sequential or Simultaneous Injection of Preformed Fibrils and AAV Overexpression of Alpha-Synuclein Are Equipotent in Producing Relevant Pathology and Behavioral Deficits. J Parkinsons Dis 12, 1133–1153, doi:10.3233/JPD-212555 (2022). 35213388 PMC9198765

[36] Thakur P. Modeling Parkinson's disease pathology by combination of fibril seeds and alpha-synuclein overexpression in the rat brain. Proc Natl Acad Sci U S A 114, E8284–E8293, doi:10.1073/pnas.1710442114 (2017). 28900002 PMC5625925

[37] Duffy M. F. Lewy body-like alpha-synuclein inclusions trigger reactive microgliosis prior to nigral degeneration. J Neuroinflammation 15, 129, doi:10.1186/s12974-018-1171-z (2018). 29716614 PMC5930695

[38] Froula J. M. Defining alpha-synuclein species responsible for Parkinson's disease phenotypes in mice. J Biol Chem 294, 10392–10406, doi:10.1074/jbc.RA119.007743 (2019). 31142553 PMC6615698

[39] Polinski N. K. Best Practices for Generating and Using Alpha-Synuclein Pre-Formed Fibrils to Model Parkinson's Disease in Rodents. J Parkinsons Dis 8, 303–322, doi:10.3233/JPD-171248 (2018). 29400668 PMC6004926

[40] Chatterjee D. Enhanced CNS transduction from AAV.PHP.eB infusion into the cisterna magna of older adult rats compared to AAV9. Gene Ther 29, 390–397, doi:10.1038/s41434-021-00244-y (2022). 33753910 PMC9203269

[41] Tyson T., Steiner J. A. & Brundin P. Sorting out release, uptake and processing of alpha-synuclein during prion-like spread of pathology. J Neurochem 139 Suppl 1, 275–289, doi:10.1111/jnc.13449 (2016). 26617280 PMC4958606

[42] Vargas J. Y., Grudina C. & Zurzolo C. The prion-like spreading of alpha-synuclein: From in vitro to in vivo models of Parkinson's disease. Ageing Res Rev 50, 89–101, doi:10.1016/j.arr.2019.01.012 (2019). 30690184

[43] Chatterjee D. & Kordower J. H. Immunotherapy in Parkinson's disease: Current status and future directions. Neurobiol Dis 132, 104587, doi:10.1016/j.nbd.2019.104587 (2019). 31454546

[44] Kharel S. & Ojha R. Future of Monoclonal Antibody Therapy in Parkinson's Disease. Ann Neurosci 30, 8–10, doi:10.1177/09727531221136349 (2023). 37313331 PMC10259150

[45] Karpowicz R. J. Jr., Trojanowski J. Q. & Lee V. M. Transmission of alpha-synuclein seeds in neurodegenerative disease: recent developments. Lab Invest 99, 971–981, doi:10.1038/s41374-019-0195-z (2019). 30760864 PMC6609465

[46] Roshanbin S. Reduction of alphaSYN Pathology in a Mouse Model of PD Using a Brain-Penetrating Bispecific Antibody. Pharmaceutics 14, doi:10.3390/pharmaceutics14071412 (2022). PMC9318263 35890306

[47] Spencer B. alpha-synuclein conformational antibodies fused to penetratin are effective in models of Lewy body disease. Ann Clin Transl Neurol 3, 588–606, doi:10.1002/acn3.321 (2016). 27606342 PMC4999592

[48] Sulzer D. & Edwards R. H. The physiological role of alpha-synuclein and its relationship to Parkinson's Disease. J Neurochem 150, 475–486, doi:10.1111/jnc.14810 (2019). 31269263 PMC6707892

[49] Fusco G. Structural basis of membrane disruption and cellular toxicity by alpha-synuclein oligomers. Science 358, 1440–1443, doi:10.1126/science.aan6160 (2017). 29242346

[50] Man W. K. The docking of synaptic vesicles on the presynaptic membrane induced by alpha-synuclein is modulated by lipid composition. Nat Commun 12, 927, doi:10.1038/s41467-021-21027-4 (2021). 33568632 PMC7876145

[51] Middleton E. R. & Rhoades E. Effects of curvature and composition on alpha-synuclein binding to lipid vesicles. Biophys J 99, 2279–2288, doi:10.1016/j.bpj.2010.07.056 (2010). 20923663 PMC3042580

[52] Burre J. Alpha-synuclein promotes SNARE-complex assembly in vivo and in vitro. Science 329, 1663–1667, doi:10.1126/science.1195227 (2010). 20798282 PMC3235365

[53] Lou X., Kim J., Hawk B. J. & Shin Y. K. alpha-Synuclein may cross-bridge v-SNARE and acidic phospholipids to facilitate SNARE-dependent vesicle docking. Biochem J 474, 2039–2049, doi:10.1042/BCJ20170200 (2017). 28495859 PMC5772654

[54] Wang L. alpha-synuclein multimers cluster synaptic vesicles and attenuate recycling. Curr Biol 24, 2319–2326, doi:10.1016/j.cub.2014.08.027 (2014). 25264250 PMC4190006

[55] Abeliovich A. Mice lacking alpha-synuclein display functional deficits in the nigrostriatal dopamine system. Neuron 25, 239–252, doi:10.1016/s0896-6273(00)80886-7 (2000). 10707987

[56] Dauer W. Resistance of alpha -synuclein null mice to the parkinsonian neurotoxin MPTP. Proc Natl Acad Sci U S A 99, 14524–14529, doi:10.1073/pnas.172514599 (2002). 12376616 PMC137916

[57] Zharikov A. Long-term RNAi knockdown of alpha-synuclein in the adult rat substantia nigra without neurodegeneration. Neurobiol Dis 125, 146–153, doi:10.1016/j.nbd.2019.01.004 (2019). 30658149 PMC6440542

[58] Zharikov A. D. shRNA targeting alpha-synuclein prevents neurodegeneration in a Parkinson's disease model. J Clin Invest 125, 2721–2735, doi:10.1172/JCI64502 (2015). 26075822 PMC4563670

[59] Benskey M. J. Silencing Alpha Synuclein in Mature Nigral Neurons Results in Rapid Neuroinflammation and Subsequent Toxicity. Front Mol Neurosci 11, 36, doi:10.3389/fnmol.2018.00036 (2018). 29497361 PMC5819572

[60] Gorbatyuk O. S. In vivo RNAi-mediated alpha-synuclein silencing induces nigrostriatal degeneration. Mol Ther 18, 1450–1457, doi:10.1038/mt.2010.115 (2010). 20551914 PMC2927065

[61] Alarcon-Aris D. Anti-alpha-synuclein ASO delivered to monoamine neurons prevents alpha-synuclein accumulation in a Parkinson's disease-like mouse model and in monkeys. EBioMedicine 59, 102944, doi:10.1016/j.ebiom.2020.102944 (2020). 32810825 PMC7452525

[62] Leavitt B. R. & Tabrizi S. J. Antisense oligonucleotides for neurodegeneration. Science 367, 1428–1429, doi:10.1126/science.aba4624 (2020). 32217715

[63] Uehara T. Amido-bridged nucleic acid (AmNA)-modified antisense oligonucleotides targeting alpha-synuclein as a novel therapy for Parkinson's disease. Sci Rep 9, 7567, doi:10.1038/s41598-019-43772-9 (2019). 31110191 PMC6527855

[64] Yang J. Exosome-mediated delivery of antisense oligonucleotides targeting alpha-synuclein ameliorates the pathology in a mouse model of Parkinson's disease. Neurobiol Dis 148, 105218, doi:10.1016/j.nbd.2020.105218 (2021). 33296726

[65] Henderson M. X. Spread of alpha-synuclein pathology through the brain connectome is modulated by selective vulnerability and predicted by network analysis. Nat Neurosci 22, 1248–1257, doi:10.1038/s41593-019-0457-5 (2019). 31346295 PMC6662627

[66] Chuapoco M. R. Adeno-associated viral vectors for functional intravenous gene transfer throughout the non-human primate brain. Nat Nanotechnol 18, 1241–1251, doi:10.1038/s41565-023-01419-x (2023). 37430038 PMC10575780

[67] Burtscher J., Copin J. C., Sandi C. & Lashuel H. A. Pronounced alpha-Synuclein Pathology in a Seeding-Based Mouse Model Is Not Sufficient to Induce Mitochondrial Respiration Deficits in the Striatum and Amygdala. eNeuro 7, doi:10.1523/ENEURO.0110-20.2020 (2020). PMC7438057 32487763

[68] Zhang Y. Rapid single-step induction of functional neurons from human pluripotent stem cells. Neuron 78, 785–798, doi:10.1016/j.neuron.2013.05.029 (2013). 23764284 PMC3751803

[69] Hockemeyer D. A drug-inducible system for direct reprogramming of human somatic cells to pluripotency. Cell Stem Cell 3, 346–353, doi:10.1016/j.stem.2008.08.014 (2008). 18786421 PMC4097107

[70] Fasano C. A. Bmi-1 cooperates with Foxg1 to maintain neural stem cell self-renewal in the forebrain. Genes Dev 23, 561–574, doi:10.1101/gad.1743709 (2009). 19270157 PMC2658524

[71] Benskey M. J., Sandoval I. M. & Manfredsson F. P. Continuous Collection of Adeno-Associated Virus from Producer Cell Medium Significantly Increases Total Viral Yield. Hum Gene Ther Methods 27, 32–45, doi:10.1089/hgtb.2015.117 (2016). 26863210

[72] Sandoval I. M., Kuhn N. M. & Manfredsson F. P. Multimodal Production of Adeno-Associated Virus. Methods Mol Biol 1937, 101–124, doi:10.1007/978-1-4939-9065-8_6 (2019). 30706392

